# Epiregulin promotes trophoblast epithelial–mesenchymal transition through poFUT1 and O‐fucosylation by poFUT1 on uPA

**DOI:** 10.1111/cpr.12745

**Published:** 2019-12-30

**Authors:** Xinyuan Cui, Hao Wang, Yaqi Li, Tianhong Chen, Shuai Liu, Qiu Yan

**Affiliations:** ^1^ Liaoning Provincial Core Lab of Glycobiology and Glycoengineering College of Basic Medical Sciences Dalian Medical University Dalian China

## Abstract

**Objectives:**

The transformation of cytotrophoblasts into mesenchymal‐like extravillous trophoblasts is necessary for successful embryo implantation, and the inadequate transformation may cause abortion. Epiregulin, which is a new growth factor, plays important roles in the reproductive processes. The glycosylation of many proteins in reproduction processes is critical. Protein O‐fucosyltransferase 1 (poFUT1) is the key enzyme for the biosynthesis of O‐fucosylation on the specific glycoproteins. Urokinase‐type plasminogen activator (uPA) contains O‐fucosylated domain on Thr^18^. However, the functions of epiregulin and poFUT1 in the trophoblast epithelial–mesenchymal transition (EMT) process, the regulatory mechanism of epiregulin on poFUT1 and the resulting O‐fucosylated uPA remain unclear.

**Materials and methods:**

We employed ELISA and Western blot to detect serum levels of epiregulin and poFUT1 from non‐pregnancy women, pregnancy women and abortion patients. Using two trophoblast cell lines and a mouse pregnancy model, we investigated the underlying mechanisms of epiregulin and poFUT1 in trophoblast EMT process.

**Results:**

Serum levels of epiregulin and poFUT1 were higher in pregnant women compared with non‐pregnant women, and their levels were significantly decreased in abortion patients compared with pregnant women. The results showed that epiregulin upregulated poFUT1 expression and increased O‐fucosylation on uPA, which further activated the PI3K/Akt signalling pathway, facilitating EMT behaviour of trophoblast cells and embryo implantation in the mouse pregnant model.

**Conclusions:**

Level of epiregulin and poFUT1 is lower in abortion patients than early pregnancy women. Epiregulin promotes trophoblast EMT through O‐fucosylation on uPA catalysed by poFUT1. Epiregulin and poFUT1 may be suggested as the potential diagnostic biomarkers and useful treatment targets for abortion.

## 1 INTRODUCTION

Embryo implantation is the process by which the mature blastocyst successfully attaches to the receptive endometrium, followed by the establishment of the placenta, which plays a crucial role in reproduction.^1^, ^2^ The trophoblasts are derived from trophectoderm cells in the blastocyst, and differentiate into syncytiotrophoblasts and cytotrophoblasts, which participate in the nutrients and gas exchange for the foetus. The extravillous trophoblast (EVT) cells originated from the cell column of villi act as a diver to lead embryo to invade into the maternal decidua, followed by the placentation and remodelling of the uterine spiral arteries.^3^, ^4^, ^5^ During the differentiation process of cytotrophoblasts into EVT cells, cytotrophoblasts undergo epithelial–mesenchymal transition (EMT) and secret extracellular matrix (ECM) degradation‐related proteins (MMP‐2/MMP‐9) to facilitate embryo to seeding, further implantation. Inappropriate or shallow invasion of trophoblast cells is the major reasons for pregnancy‐related complications, such as miscarriage, intrauterine growth restriction and preeclampsia.^6^, ^7^ Despite a large number of molecules involved in the embryo–maternal communication have been identified, the detail mechanism related to how the molecules regulate trophoblasts cell migration and invasion capacity requires further study.

Epiregulin belongs to the epidermal growth factor (EGF) family. It contains 46 amino acids and is secreted in soluble form. Epiregulin usually binds to the EGF receptor (ErbB1 and ErbB4).^7^, ^8^ The increasing evidence indicates that epiregulin plays important roles in the reproductive processes. The human epiregulin is mainly found in the placenta and uterus at the site of blastocyst implantation,^9^ and Haengseok Song also reported that epiregulin expressed in the luminal epithelium and the underlying stroma surrounding the blastocysts during the attachment reaction.^10^ These studies indicate that epiregulin is involved in embryo implantation. Dysregulation of the EGF family in the uterus induces implantation failure. HB‐EGF affects blastocyst activities related to implantation.^11^ Furthermore, the EGF signalling cascades are necessary in regulating trophoblast differentiation, and its disruption could cause perinatal diseases, such as preeclampsia and intrauterine growth restriction. However, whether abnormal epiregulin can influence blastocyst implantation remains unclear.

Glycosylation is a post‐translational modification of proteins, which plays a crucial role in the reproduction. Fucosylation, a typical type of glycosylation, is classified into two forms, N‐fucosylation and O‐fucosylation. Fucosyltransferases, including N‐fucosyltransferases (FUTs) and protein O‐fucosyltransferases (poFUT1/poFUT2), transfer fucose residue to the acceptor by N‐linkage or O‐linkage, respectively.^12^, ^13^, ^14^ poFUT1 adds O‐linked fucose to the folding EGF repeats containing the consensus sequence C^2^‐X‐X‐X‐X‐(S/T)‐C^3^.^15^ Fucosylation and fucosyltransferases are critical for reproduction, and aberrant fucosylation of proteins is associated with the reproduction disorders. N‐glycosylation of embryo proteins is required to facilitate embryo attachment and invasion into the epithelium.^16^, ^17^ Overexpression of fucosyltransferase 7 stimulates embryo adhesion and implantation.^18^ Leukaemia inhibitory factor promotes embryo adhesion through unregulated expression of FUT1 and Lewis Y.^19^ We have previously found that poFUT1 significantly promotes the proliferation, migration and invasion of trophoblast cells in vitro. However, the role of epiregulin on poFUT1/O‐fucosylation in the process of trophoblast invasion remains unknown.

Urokinase‐type plasminogen activator (uPA) is a single‐polypeptide‐chain glycosylated zymogen that consists of three domains: a growth factor domain (GFD, 1‐49 amino acids), a kringle domain (KD, 50‐131 amino acids) and a serine protease domain (159‐411 amino acids). After binding uPAR, pro‐uPA zymogen becomes active uPA (GFD), which in turn cleaves and activates MMPs and degrades ECM. The GFD domain of uPA contains an EGF‐like domain, which can be fucosylated on Thr^18^.^13^, ^20^, ^21^ The uPA/uPAR indicates the different downstream intracellular signalling pathways, such as PI3K, FAK and Rac signalling pathways,^22^, ^23^, ^24^ thus regulating cell proliferation, migration, invasion and angiogenesis, etc For example, uPA/uPAR system is involved in the male reproductive. uPA can stimulate sperm mobility and promote fertilization.^25^, ^26^ uPA is also secreted by trophoblast and foetal membrane cells and endovascular cells, which facilitates trophoblast invasion into the uterine endometrium.^26^ However, lower uPA may prevent trophoblast invasion, and possibly as a preeclampsia marker.^27^, ^28^ These findings suggest that trophoblast uPA/uPAR is a critical determinant of embryo implantation. uPA contains the fucosylated EGF domain in human SaOS‐2 osteosarcoma cells and U‐937 lymphoma cells. However, the relationship between uPA, especially the O‐fucosylation of uPA, and miscarriage is still unclear.

In the present study, the serum level of epiregulin and poFUT1 in pregnancy women and abortion patients were examined, and the decreased levels of epiregulin and poFUT1 were associated with abortion. The upregulation of poFUT1 by epiregulin increases the fucosylation of uPA, which activates uPA/uPAR‐mediated PI3K/Akt signalling pathway, and facilitated EMT of trophoblast cells.

## 2 MATERIALS AND METHODS

### 2.1 Serum and tissue samples

All experimental protocols for human study were in accordance with the approved guidelines by the Institutional Review Boards of Dalian Medical University. The villi tissues and serum samples of women at the age of 25‐35 were obtained from The Second Affiliated Hospital of Dalian Medical University (Dalian, China). The pregnant women (n = 20) and abortion patients (n = 20) were confirmed by ultrasound detection at 6‐10 gestational weeks. Healthy villi tissues were obtained from women undergoing legal abortion for non‐medical reasons. The abortion tissues were from the first‐trimester patients who underwent induced abortion, with serum progesterone level less 25 ng/mL.

### 2.2 Cell culture

The human trophoblastic HTR‐8/SVneo and JAR cell lines were obtained from the American Type Culture Collection. Cells were cultured in DMEM/F12 (HyClone) conditional medium supplemented with 10% FBS (Gibco) and 1% penicillin–streptomycin. The medium was renewed every 2‐3 days. Cells were maintained in a humidified atmosphere containing 5% CO_2_ at 37°C.

### 2.3 Real‐time PCR

Cells were treated with RNAiso Plus reagent (Takara) for RNA extraction, and the PrimeScript RT Reagent Kit with a gDNA Eraser kit (Takara) was used to synthesize cDNA. SYBR Premix Ex Taq (Takara) was used for q‐PCR. Primer sequences were as followings: GAPDH (forward) 5′‐ATGGGGAAGGTGAAGGTCG‐3′, (reverse) 5′‐GGGGTCATTGATGGCAACAATA‐3′, poFUT1 (forward) 5′‐CAGCGAAGCCCAGATAAGAAG‐3′, (reverse) 5′‐CTGTAGGAAGCTCTGAAGGAAAT‐3′, E‐cadherin (forward) 5′ ‐CAACGACCCAACCCAAGAA‐3′, (reverse) 5′‐CCGAAGAAACAGCAAGAGCA‐3′, N‐cadherin (forward) 5′‐AAAGAACGCCAGGCCAAAC‐3′, (reverse) 5′‐GGCATCAGGCTCCACAGTGT‐3′. The reactions were performed using the Applied Biosystems 7500 Fast Real‐time PCR System (Life Technologies). Quantified data were normalized to those of GAPDH, and the relative quantity was calculated using the 2^−ΔΔCT^ method.

### 2.4 Transfection

HTR‐8/SVneo cells and JAR were seeded onto the plates. When cells reached 70% confluence, scramble or poFUT1 siRNA: 5′‐GGUCUACGUUGCUACUGAUTT‐3′ (sense), 5′‐AUCAGUAGCAACGUAGACCTT‐3′ (antisense) (GenePharma) was transiently transfected into the cells using Lipofectamine 2000 reagent (Invitrogen) according to the manufacturer's instructions. The transfection reagent was removed 6 hours later. Total protein and RNA were collected after 48 hours.

### 2.5 Western blot

To prepare whole‐cell protein lysates, cells at 90% confluence were washed in PBS before incubation with RIPA lysis buffer. Equal protein was loaded onto 10% SDS‐PAGE gels, transferred onto nitrocellulose membranes and blocked with TTBS containing 5% fat‐free dry milk. The membranes were incubated at 4°C overnight with the primary antibodies: uPA and uPAR (Abcam), p‐c‐FOS (Ser^32^), p‐c‐JUN (Ser^73^), c‐FOS, c‐JUN, Akt, p‐Akt (Tyr^308^), PDK and p‐PDK (Ser^241^) (Cell Signaling Technology) and CK‐7, vimentin, N‐cadherin, E‐cadherin, poFUT1, HLA‐G and GAPDH (Proteintech). Next, the membranes were incubated with HRP‐conjugated goat anti‐rabbit IgG, HRP‐conjugated goat anti‐mouse IgG or HRP‐conjugated streptavidin for 1 hour. An enhanced chemiluminescence detection system (Bio‐Rad) was used to visualize immunoreactive bands.

### 2.6 Immunofluorescent staining

Cover slips (cells) or frozen slices (tissues) were fixed in 4% paraformaldehyde or cold acetone for 30 minutes, followed by blocking with 1% goat serum (Beyotime) for 2 hours. Next, the cover slips or slices were incubated with different antibodies at proper dilutions: poFUT1 was applied at 4°C overnight followed by incubation with the FITC or TRITC‐conjugated second antibody for 1 hour. After counterstaining with DAPI (blue) for 5 minutes, anti‐fade solution (Beyotime) was added to mount the coverslips or slices before imaging under the fluorescent microscope (Olympus).

### 2.7 Matrigel invasion

For Matrigel invasion assay, Transwell inserts (Costar) containing polycarbonate filters with 8‐μm pores were precoated with 50 μL of 1 mg/mL Matrigel matrix. Cells (1.0 × 10^5^) in serum‐free medium were plated in the upper chamber, whereas medium with 10% FBS was added to the lower chamber. After incubating for 24 hours, the cells on the Matrigel side of the inserts were removed by the cotton swab. The inserts were fixed in methanol and stained with crystal violet dye. The number of invaded cells attached to the other side of the insert was counted under a light microscope (Olympus) in five random fields. Three independent experiments were performed.

### 2.8 Labelling and detection of glycoproteins in cell extracts

Cells seeded on culture dish were treated with/without test sugars (200 μmol/L 6‐alkynyl fucose) in growth medium at 37°C. After 3 days, cell extracts were prepared by resuspending the cells in 200 μL lysis buffer. Protein extract (1 mg/mL) was labelled for 1 hour at room temperature (azido‐probe). The Click‐iT^®^ Protein Reaction Buffer Kit includes the reagents required to perform the click reaction on proteins labelled with an azide‐tagged biomolecule. Labelled protein lysate was resolved by SDS/PAGE.

### 2.9 Microscopic analysis of fluorescent labelling O‐glycosylation in cells

HTR‐8/SVneo and JAR cells were seeded onto six‐well plates containing glass coverslips. Growth medium was supplemented with 200 μmol/L 6‐alkynyl fucose. After growing for 3 days, cells on coverslips were fixed and permeabilized with acetone for 10 minutes then subjected to the probe labelling reaction. Subsequently, the fixed and labelled cells were stained with TRITC‐conjugated streptavidin for 1 hour. DAPI was used to stain nuclei. Fluorescent images were captured by fluorescent microscope.

### 2.10 Immunoprecipitation

Immunoprecipitation was performed with the Dynabeads^®^ Protein G Kit (Life Technologies) by following the standard procedures. Briefly, total protein lysates were added to the Dynabeads‐uPA or uPAR antibody (Ab) complex for 30 minutes at room temperate, followed by washing 3 times. Subsequently, the Dynabeads‐Ab‐protein complex was mixed with elution and lysis buffer, and incubated 15 minutes at 70°C to denature the proteins.

### 2.11 Mouse embryos collection

All animal experiments from this paper were approved by the Animal Ethics Committee of Dalian Medical University. The species of Kunming mice (7‐12 weeks) were from the Laboratory Animal Center of Dalian Medical University, China. The mice were feeding in stable environmental conditions (temperature 20‐25°C; humidity: 60%). After mating, when the female mice were found a vaginal plug in the next morning, it was defined as pregnant day 1. On the day 3.5, the pregnant mice were euthanized by cervical dislocation and cut out the uteri. In order to get embryos, the uterine cavity was flushed with warm PBS (without Ca^2+^ and Mg^2+^). Next, embryos were placed in 96‐well plates and cultured at 37°C under 5% CO_2_ in humidified air according to the standard procedures.

### 2.12 Mouse embryo transfer

The pseudopregnant recipient females used for embryo transfer were obtained by natural mating with vasectomized males. The seminal secretions produced by a sterile male were required for the uterus to become receptive to the transferred embryos. To obtain a recipient, 2 females of 7‐12 weeks of age were placed with a vasectomized male in the afternoon. The following morning, females were checked for the presence of a vaginal copulation plug, a clump of coagulated proteins from the male seminal fluid. The day of vaginal plug detection was considered to be day 0.5. Different treatments of mouse morulae or blastocysts were transferred to the bilateral uterus of pseudopregnant recipient female at days 2.5‐3. The uterus was removed from the recipient 8 days after embryo transfer.

### 2.13 Statistical analysis

GraphPad Prism^®^ (GraphPad Software Inc) was used for statistical analysis. All experiments were performed at least 3 independent times, and the data were shown as means ± SEM. For the analysis of difference between groups, independent samples *t* test was performed. The Spearman correlation analysis was used to analyse the relationship between epiregulin and poFUT1 in the serum. The receiver operating characteristic curve was applied to evaluate the diagnostic value of the epiregulin and poFUT1. The statistical significance was indicated as the follows: *P < .05, **P < .01 and ***P < .001.

## 3 RESULTS

### 3.1 Low epiregulin, poFUT1 and uPA levels in abortion patients

**Figure 1:**
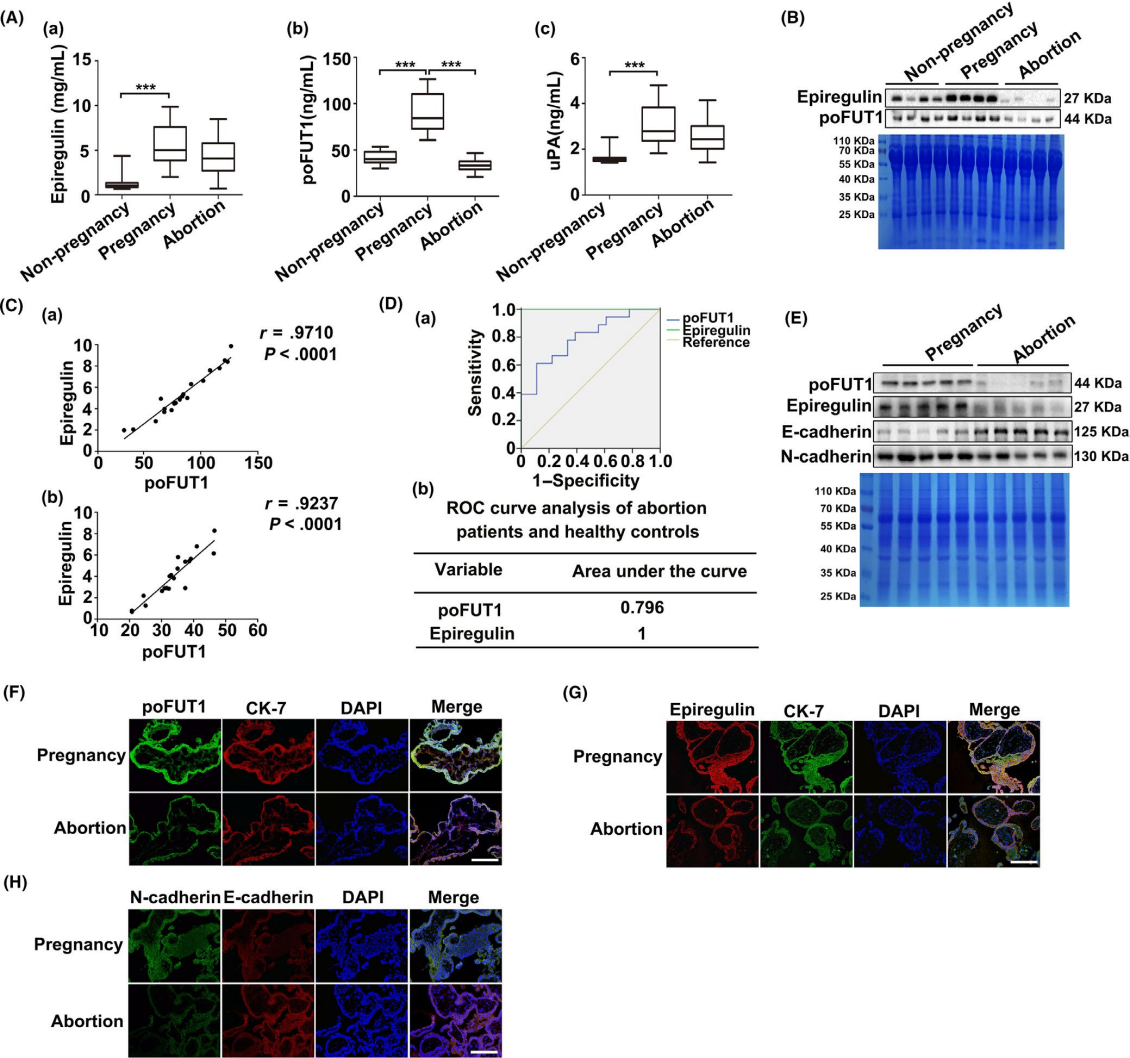
Epiregulin and poFUT1 levels in pregnant and abortion women. Serum levels of epiregulin, poFUT1 and uPA in non‐pregnancy, pregnancy and abortion patients detected by ELISA (A) and Western blot (B). C, Correlation analysis between Epiregulin and poFUT1 expression in serum of pregnancy and abortion patients. D, ROC curve analysis of the diagnostic between epiregulin and poFUT1. E, poFUT1, epiregulin, E‐cadherin and N‐cadherin expression in villi tissues were detected by Western blot. F, Immunofluorescent staining of poFUT1 (green) in tissues. CK‐7 (red) was stained as the villi marker. Nuclei were stained with the DAPI (blue). G, Immunofluorescent staining of epiregulin (red) in tissues. CK‐7 (green) was stained as the villi marker. H, Villi tissues were analysed for N‐cadherin (green) and E‐cadherin (red) by immunofluorescent staining. Scale bars, 100 μm. CBB, Coomassie Brilliant Blue; poFUT1, protein O‐fucosyltransferase 1; ROC, receiver operating characteristic; uPA, urokinase‐type plasminogen activator

We first performed ELISA and Western blot to determine the levels of epiregulin, poFUT1 and uPA in the serum from non‐pregnant, pregnant and abortive women. As shown by ELISA (Figure 1), the levels of epiregulin, poFUT1 and uPA were decreased in the serum of abortion patients compared with pregnant women (Figure 1A). Western blot also showed that epiregulin and poFUT1 levels were higher in the pregnant women than in the abortion patients (Figure 1B). It was worth noting that there was a positive correlation between the epiregulin and poFUT1 expression levels in the serum of pregnant women (*r* = .9710) and abortion patients (*r* = .9237) (Figure 1C). We further evaluated the diagnostic value of poFUT1 and epiregulin by receiver operating characteristic curve (ROC) analysis and determined that epiregulin (area under the curve: 1) and poFUT1 (area under the curve: 0.796) were potential diagnostic markers for abortion patients (Figure 1D). We compared the expression of epiregulin and poFUT1 in the villi of pregnant women and abortion patients (Figure 1E). Immunofluorescence staining showed that epiregulin and poFUT1 were localized in villous trophoblast cells, and both stainings were stronger in normal pregnant women than in abortion patients (Figure 1F,G). We further explored the expression of EMT markers (N‐cadherin and E‐cadherin) in the villi of pregnant women and abortion patients by immunoblotting and immunostaining (Figure 1H). The staining showed higher N‐cadherin and lower E‐cadherin levels in pregnant women than in abortion patients (Figure 1D‐F). These results indicate that low level of epiregulin, poFUT1 and uPA is related to women abortion.

### 3.2 Epiregulin promotes trophoblast cell invasion and migration through EMT

**Figure 2:**
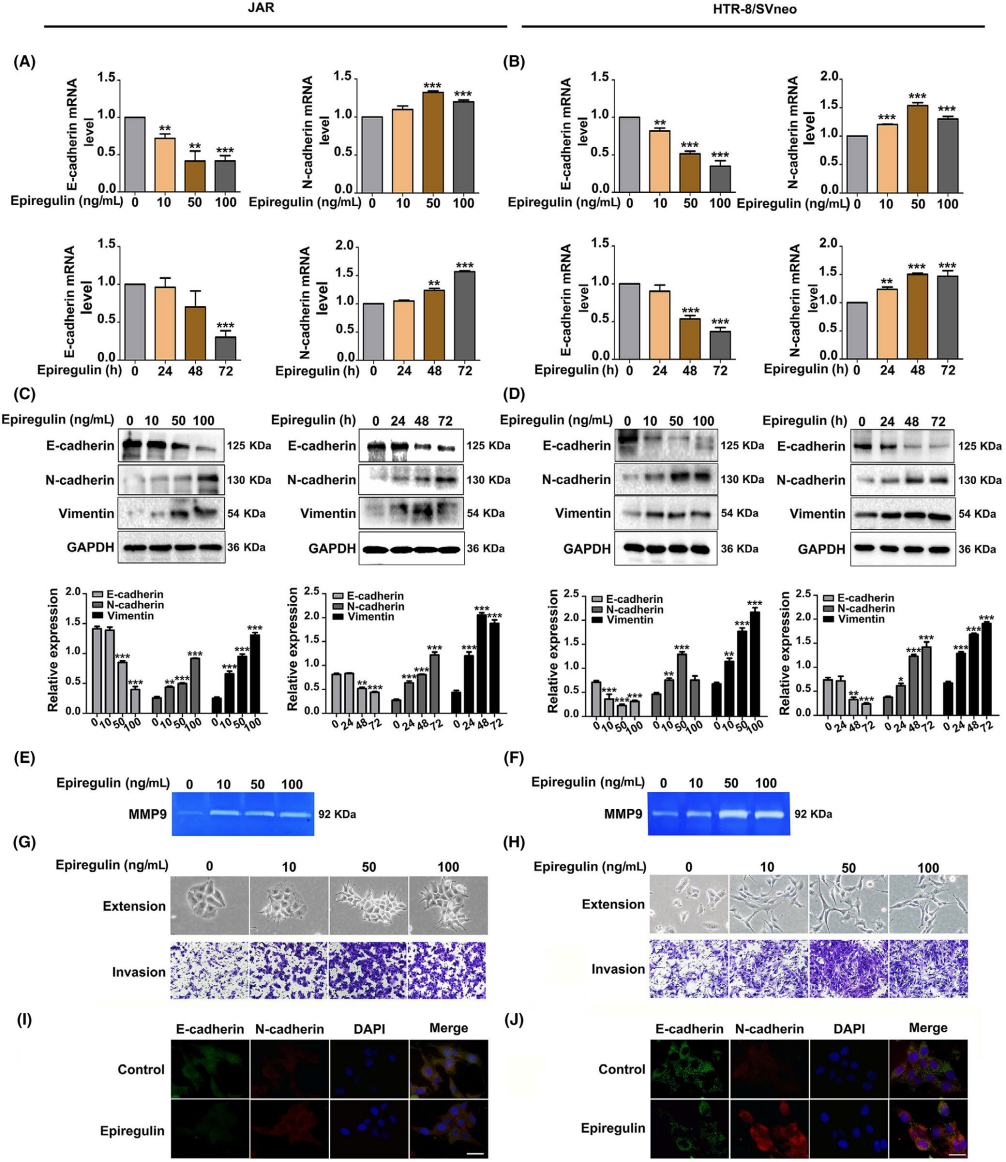
Epiregulin promotes EMT of human trophoblast cells. A, JAR and (B) HTR‐8/SVneo cells were treated with epiregulin at different concentrations (0, 10, 50, 100 ng/mL) and with 50 ng/mL epiregulin for different times (0, 24, 48, 72 h). Total RNA was analysed for E‐cadherin and N‐cadherin expression by RT‐PCR, with GAPDH serving as the internal control. C, JAR and (D) HTR‐8/SVneo cell lysates were prepared for immunoblotting analysis for E‐cadherin, N‐cadherin and vimentin, and GAPDH served as the internal control. E and F, Analysis of MMP9 activity by gelatine zymography. G and H, Cell extension, and invasive ability were examined by Transwell invasion assays. I, JAR and (J) HTR‐8/SVneo cells were analysed for E‐cadherin (green) and N‐cadherin (red) by immunofluorescent staining. DAPI was used for nuclear staining. Scale bars: 50 μm. The statistical analysis was shown: *P < .05; **P < .01; ***P < .001. EMT, epithelial–mesenchymal transition

To evaluate the effects of epiregulin on the migration and invasion capacities of trophoblast cells, we treated cells with different concentrations of epiregulin (0, 10, 50, 100 ng/mL) and for different time periods (0, 24, 48, 72 hours). RNA and protein samples were collected. The results showed the downregulation of E‐cadherin and upregulation of N‐cadherin and vimentin by real‐time PCR (Figure 2A,B). The effect of epiregulin on EMT markers was further confirmed by Western blot (Figure 2C,D) and immunofluorescence staining (Figure 2I,J). The invasive potential of HTR‐8/SVneo and JAR cells was studied by assaying the activities of MMP2 and MMP9 in the culture medium using gelatine zymography; the results indicated that MMP9 activity was increased by epiregulin treatment (Figure 2E,F). To detect alterations in the invasion abilities after epiregulin treatment, Transwell assays were applied. Epiregulin significantly promoted cell extension, invasion ability compared with the control cells (Figure 2G,H). These results suggest that epiregulin promotes the invasion potential of HTR‐8/SVneo and JAR trophoblastic cells.

### 3.3 Epiregulin upregulates poFUT1 expression through AP‐1

**Figure 3:**
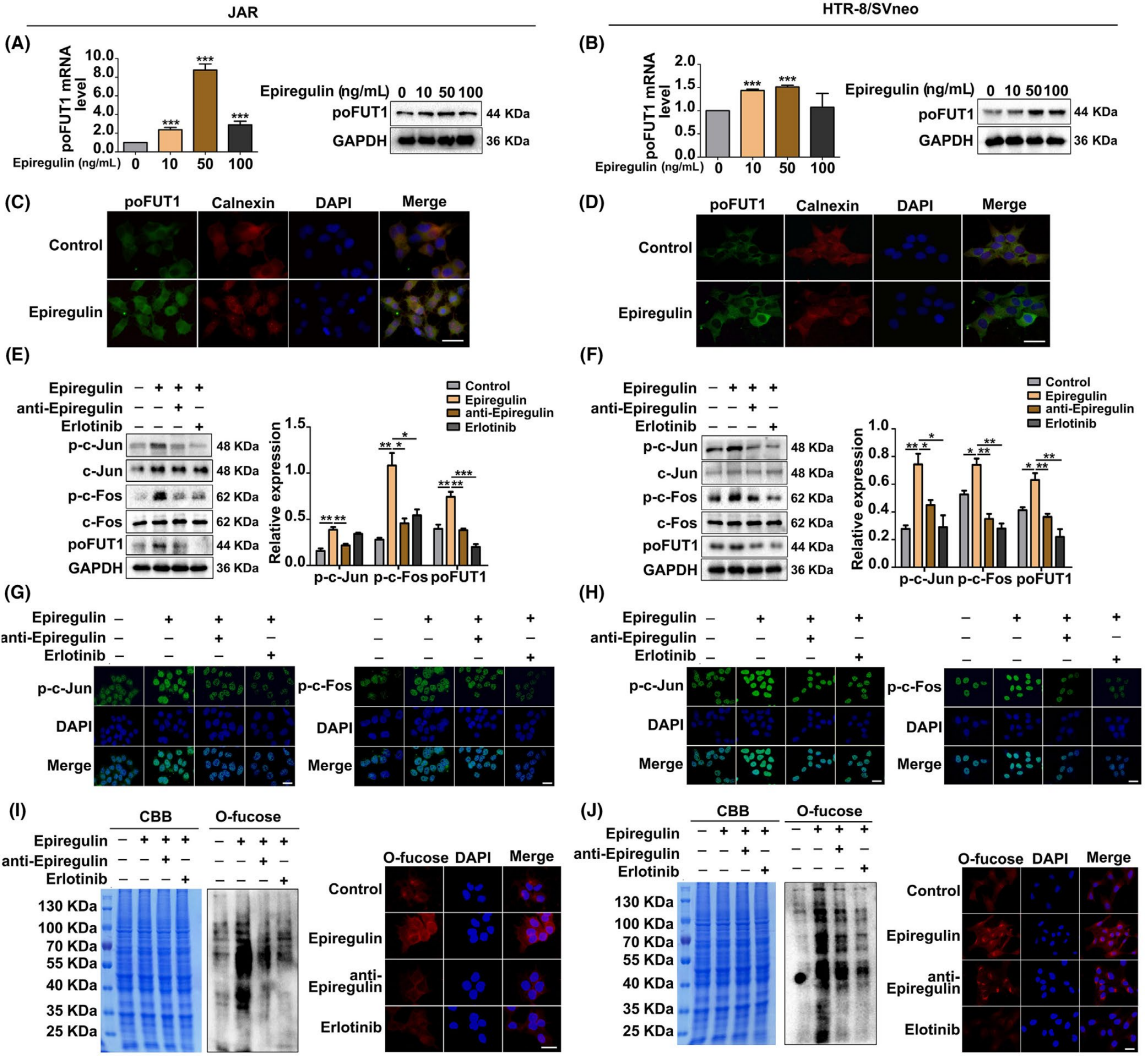
Epiregulin increases poFUT1 and O‐fucosylation expression through active transcription factor AP‐1. poFUT1 expression was detected by RT‐PCR and Western blot in (A) JAR and (B) HTR‐8/SVneo cells after treatment with epiregulin (0, 10, 50, 100 ng/mL) for 48 h. Immunofluorescence staining of poFUT1 in trophoblast cells (C, D). Green: poFUT1. Red: calnexin. Blue: DAPI. Analysis of levels of phosphorylated and non‐phosphorylated c‐Fos/c‐Jun and poFUT1 by Western blot (E, F) and immunofluorescence (G, H) in trophoblast cells treated with epiregulin (50 ng/mL) for 48 h, anti‐epiregulin antibody and erlotinib (EGFR inhibitor). Lectin blotting and lectin staining (I, J) were used to analyse the effect of poFUT1 on O‐fucosylation biosynthesis in trophoblasts by treatment with epiregulin (50 ng/mL) for 48 h, anti‐epiregulin antibody or erlotinib (EGFR inhibitor). Scale bars: 50 μm. The statistical analysis was shown: *P < .05; **P < .01; ***P < .001. poFUT1, protein O‐fucosyltransferase 1

To explore whether epiregulin promotes EMT of trophoblast cells through poFUT1, we further detected the effect of epiregulin on poFUT1. Using real‐time PCR, Western blot and immunofluorescence staining, the effect of epiregulin on poFUT1 expression was investigated. Cells were treated with epiregulin at different concentrations of (0, 10, 50, 100 ng/mL) for 48 hours, and the expression of poFUT1 was significantly increased (Figure 3A‐D). Calnexin was used as a marker for Golgi apartment localization. To determine whether AP‐1 (c‐Fos and c‐Jun) was involved in epiregulin‐induced poFUT1 expression, cells were treated with epiregulin (50 ng/mL), epiregulin antibody and EGFR antagonist, the poFUT1 level was detected. Western blot results showed that poFUT1 expression was increased after epiregulin treatment, whereas poFUT1 expression was decreased after incubation with epiregulin antibody or EGFR antagonist. We then measured the c‐Jun and c‐Fos protein levels in the same situation, and the Western blot results showed that epiregulin promoted phosphorylation of c‐Fos and c‐Jun, whereas epiregulin antibody and EGFR antagonist lessened the enhanced effect (Figure 3E,F). Immunofluorescence staining showed similar alternations with those of Western blot analysis (Figure 3G,H). PoFUT1 is the key enzyme in O‐fucose biosynthesis. We then explored, by immunoblotting and immunostaining, whether the alteration of poFUT1 could change O‐fucose biosynthesis on cells. The results showed that epiregulin promoted O‐fucose biosynthesis, whereas epiregulin antibody and EGFR antagonist inhibited O‐fucose biosynthesis in HTR‐8/SVneo and JAR trophoblastic cells (Figure 3I,J). These data indicate that epiregulin promotes poFUT1 expression by activating c‐Fos and c‐Jun, which further increase O‐fucosylation biosynthesis.

### 3.4 poFUT1 promotes O‐fucosylation on uPA and activates the PI3K/Akt signalling pathway

**Figure 4:**
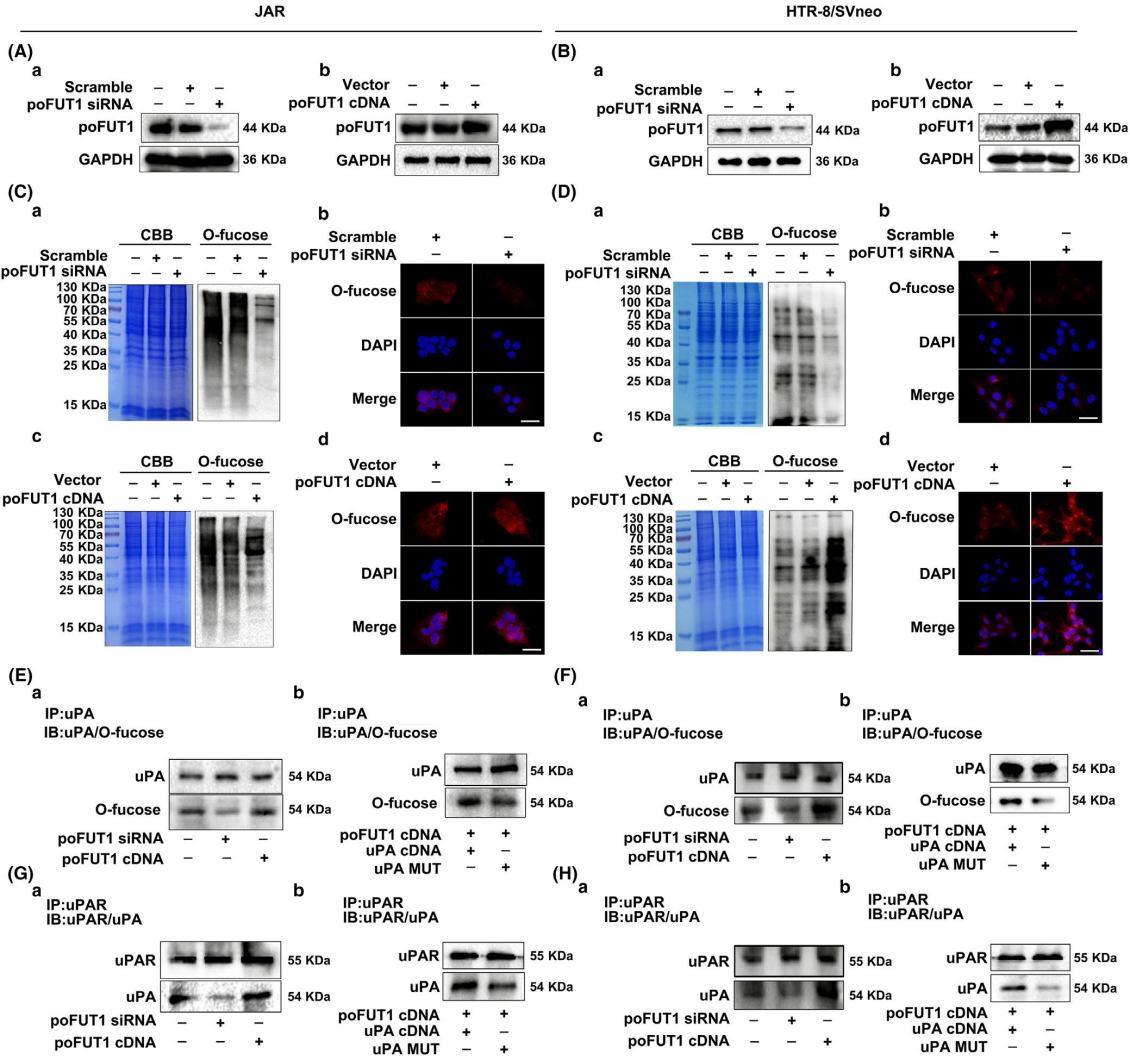
poFUT1 modifies O‐fucosylation on uPA and binding potential of uPA and uPAR. JAR and HTR‐8/SVneo cells were transfected with poFUT1 siRNA and poFUT1 cDNA. The levels of poFUT1 and O‐fucosylation were detected by Western blot and immunofluorescence staining (A‐D). The total protein lysates were immunoprecipitated with uPA antibody. The samples were immunoblotted to detect the amounts of O‐fucosylated uPA and total uPA (E, F). JAR and HTR‐8/SVneo cells were co‐transfected with uPA cDNA and poFUT1 cDNA or co‐transfected with uPA‐MUT and poFUT1 cDNA. The total protein lysates were immunoprecipitated with uPAR antibody. The samples were detected for the binding potential between uPA and uPAR (G, H). poFUT1, protein O‐fucosyltransferase 1; uPA, urokinase‐type plasminogen activator

Protein glycosylation usually influences the functions of glycoproteins. Because poFUT1 is mainly responsible for adding fucose on EGF repeats of proteins, we investigated whether poFUT1 could regulate O‐fucosylation on uPA and functions, following trophoblast cells transfected with poFUT1 siRNA or poFUT1 cDNA plasmid, Western blot detection showed that poFUT1 siRNA decreased the level of poFUT1, whereas poFUT1 cDNA increased the level of poFUT1 (Figure 4A,B). We utilized click chemistry methods to detect O‐fucosylated glycans. The results showed that poFUT1 siRNA inhibited the biosynthesis of O‐fucosylated glycans. In contrast, poFUT1 cDNA promoted O‐fucosylation in HTR‐8/SVneo and JAR trophoblast cells (Figure 4C,D). We further detected alterations in uPA O‐fucosylation using immunoprecipitation. Results showed that silencing poFUT1 by poFUT1 siRNA transfection inhibited O‐fucosylation on uPA, whereas upregulation of poFUT1 by poFUT1 cDNA plasmid enhanced O‐fucosylation on uPA (Figure 4E,F). Most importantly, poFUT1 siRNA transfection decreased the combination of uPA with uPAR, whereas poFUT1 cDNA enhanced the amount of uPA bound with uPAR (Figure 4G,H). These results demonstrate the regulatory role of poFUT1 in the biosynthesis of O‐fucosylation on uPA and the combination ability of uPA with uPAR.

**Figure 5:**
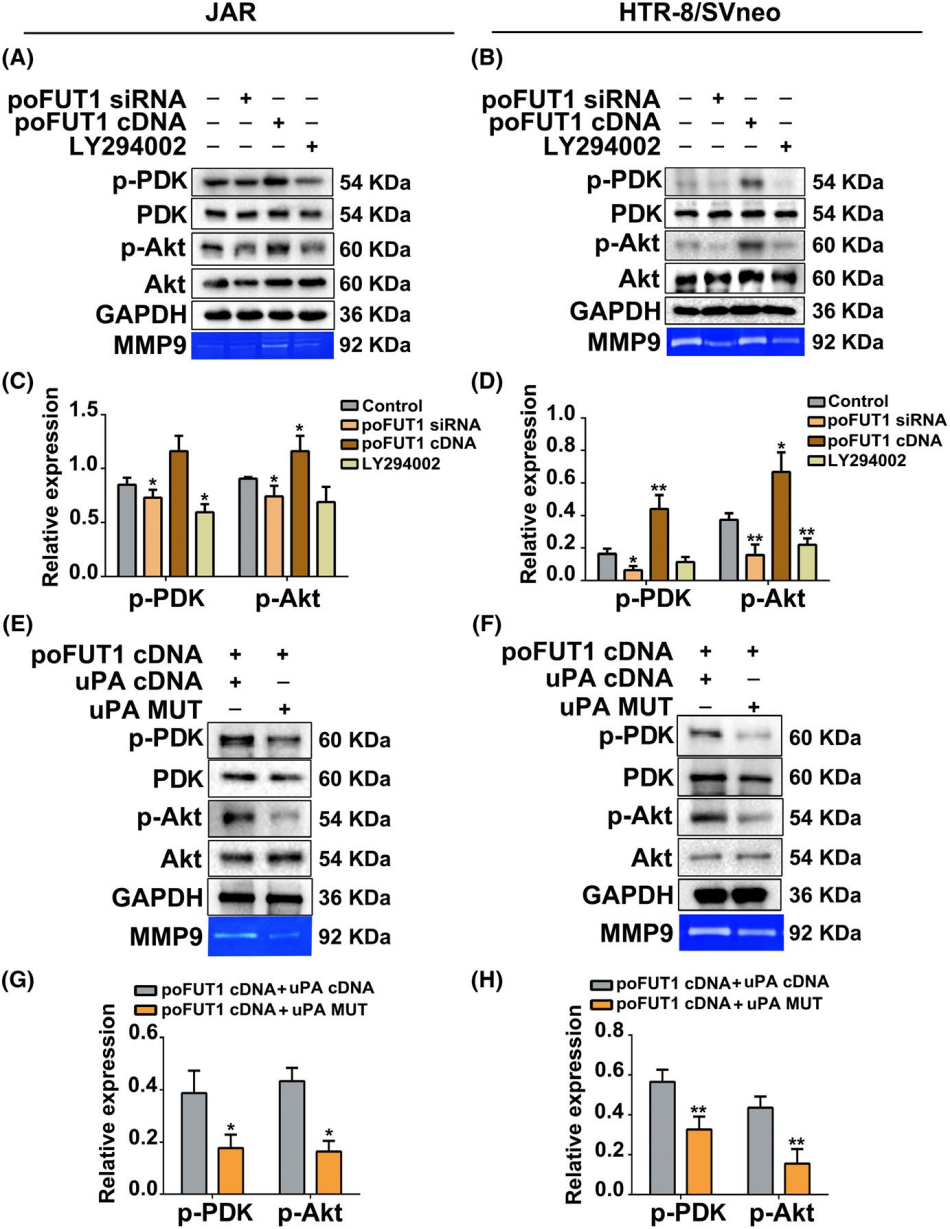
poFUT1 modifies O‐fucosylation on uPA and activates the PI3K/Akt signalling pathway. JAR and HTR‐8/SVneo cells were transfected with poFUT1 siRNA, poFUT1 cDNA and treated with PI3k inhibitor (LY294002). The expression of p‐PDK (Ser^241^), PDK, p‐Akt (Tyr^308^) and Akt in JAR and HTR‐8/SVneo cells were detected by Western blot (A, B) and statistical analysis (C, D). JAR and HTR‐8/SVneo cells were co‐transfected with uPA cDNA and poFUT1 cDNA and co‐transfected with uPA‐MUT and poFUT1 cDNA. The expression of p‐PDK (Ser^241^), PDk, p‐Akt (Tyr^308^) and Akt in JAR and HTR‐8/SVneo cells were detected by Western blot (E, F) and statistical analysis (G, H). The statistical analysis was shown: *P < .05; **P < .01. poFUT1, protein O‐fucosyltransferase 1; uPA, urokinase‐type plasminogen activator

To clarify the mechanism underlying the connection between poFUT1 and O‐fucosylation on uPA, the activation of the PI3K/Akt signalling pathway was detected. Western blot results showed that poFUT1 siRNA inactivated the PI3K/Akt signalling pathway, whereas poFUT1 cDNA enhanced PI3K/Akt signalling pathway activity and PI3K inhibitor (LY294002) suppressed it (Figure 5A‐D). Similarly, p‐PDK/p‐Akt was also decreased after cells were transfected with poFUT1 siRNA, whereas p‐PDK/p‐Akt increased after transfection with poFUT1 cDNA plasmid, and PI3K inhibitor (LY294002) reduced the expression of p‐PDK/p‐Akt. Gelatine zymography data also showed that poFUT1 siRNA resulted in a reduction in MMP9 activity (Figure 5A,B). We next asked whether O‐fucose biosynthesis on uPA could influence the PI3K/Akt signalling pathway and MMP9 activity. Western blot data showed that co‐transfection with uPA cDNA and poFUT1 cDNA stimulated the PI3K/Akt signalling pathway and MMP9 activity compared with co‐transfection with uPA mutation plasmid (uPA‐MUT) and poFUT1 cDNA (Figure 5E‐H).

### 3.5 Epiregulin promotes trophoblastic cell invasion by increasing poFUT1 expression and O‐fucosylation on uPA, which stimulates the PI3K/Akt signalling pathway and MMP9 activity

**Figure 6:**
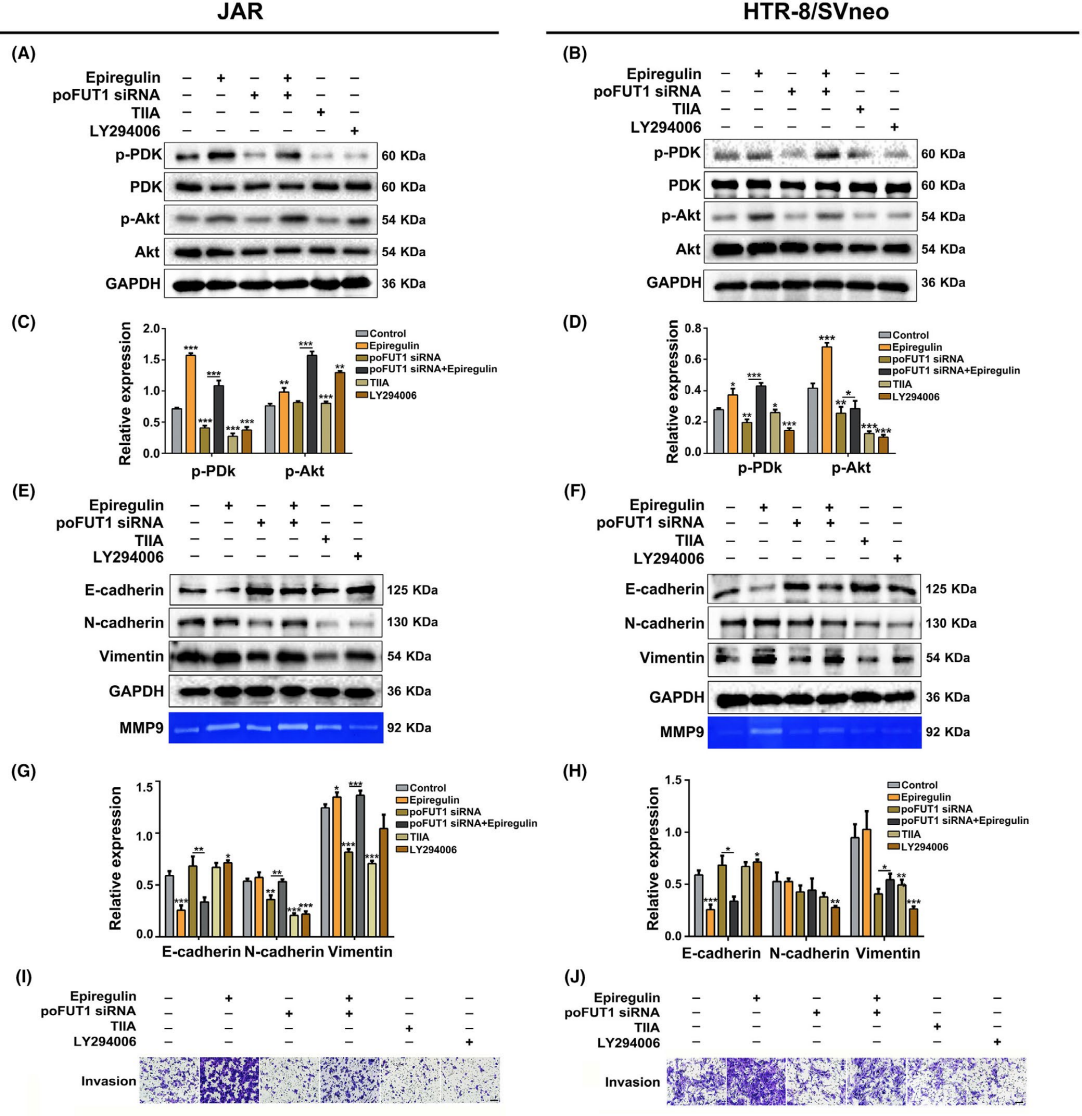
Epiregulin increases poFUT1 and O‐fucosylation on uPA and activates PI3K/Akt signalling pathway. The expression of p‐PDK (Ser^241^), PDk, p‐Akt (Tyr^308^) and Akt in JAR and HTR‐8/SVneo cells after different treatments were detected by Western blot (A‐D). The expression of E‐cadherin, N‐cadherin and vimentin in JAR and HTR‐8/SVneo cells were detected by Western blot. Analysis of MMP9 activity by gelatin zymography (E‐H). Cell invasiveness ability were examined by Transwell invasion assays (I, J). Scale bars: 50 μm. The statistical analysis was shown: *P < .05; **P < .01; ***P < .001. poFUT1, protein O‐fucosyltransferase 1; uPA, urokinase‐type plasminogen activator

The Western blot results showed that epiregulin reduced E‐cadherin expression, whereas it increased vimentin and N‐cadherin expression. Gelatine zymography data also showed that epiregulin increased MMP9 activity (Figure 6E‐H). Silenced poFUT1 was associated with an enhancement of E‐cadherin expression, whereas vimentin/N‐cadherin expression and MMP9 activity were reduced. Meanwhile, LY294002 inactivated EMT process. Similarity, TIIA, an inhibitor of AP‐1, depressed the EMT. Treatment cells with LY294002 and TIIA also inhibited the activity of MMP9 (Figure 6E‐H). Western blot results showed that poFUT1 siRNA inhibited the activation of PI3K/Akt signalling pathway, and epiregulin activated the signalling pathway (Figure 6A‐D). Transwell assays were performed to further verify that epiregulin could promote trophoblast cell invasion by targeting poFUT1 and O‐fucosylation on uPA through PI3K/Akt signalling pathway (Figure 6I,J). The results show that epiregulin promotes invasion through the activated PI3K/Akt signalling pathway, whereas poFUT1 knockdown and treatment with LY294002 and TIIA suppress invasion through PI3K/Akt signalling pathway (Figure 6).

### 3.6 Epiregulin promotes embryo implantation in vivo

**Figure 7:**
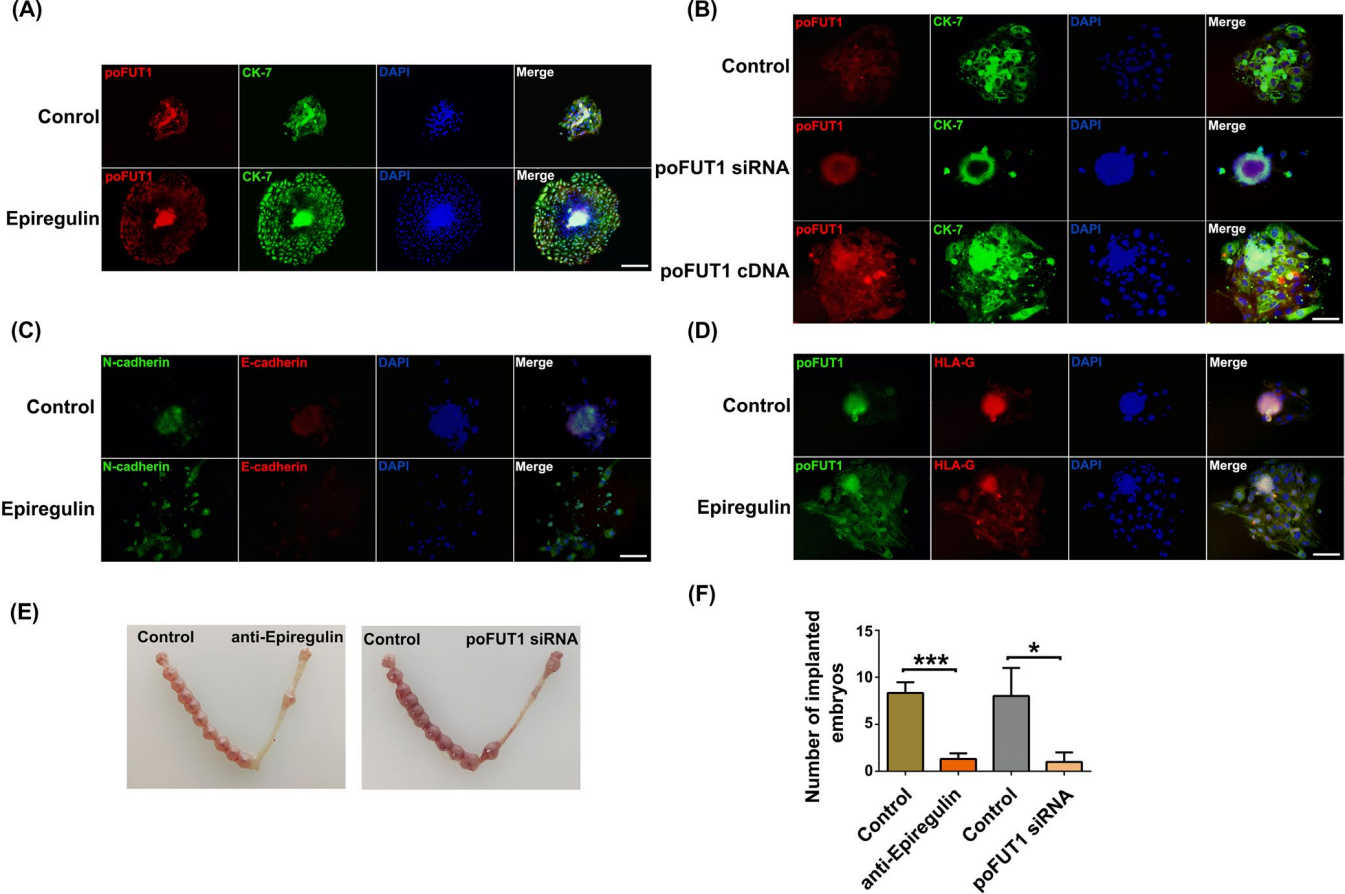
Epiregulin promotes embryo implantation in vivo. Mouse embryos were collected at PD4 and treated with epiregulin for 48 h, were analysis of poFUT1 (red) and CK‐7 (green) by immunofluorescent staining (A). The mouse embryos were transfected with poFUT1 siRNA and poFUT1 cDNA were analysis of poFUT1 (red) and CK‐7 (green) by immunofluorescent staining (B). Treated with epiregulin, mouse embryos were analysis of N‐cadherin (green) and E‐cadherin (red) by immunofluorescent staining (C). And mouse embryos were analysis of poFUT1 (green) and HLA‐G (red) by immunofluorescent staining (D). DAPI was used for nuclear staining. Scale bars: 50 μm. (E, F) Representative picture of implanted embryo at PD8 and the statistical analysis were shown, *P < .05, ***P < .001. poFUT1, protein O‐fucosyltransferase 1

To further investigate the effects of epiregulin on embryo implantation, a pregnant mouse model was employed. Mouse embryos were collected at PD3.5 and were treated with epiregulin. After incubated 48 hours, the embryos in each group were observed, and the immunofluorescent staining results showed that epiregulin promoted the expression of poFUT1 and invasion potential of mouse embryos (Figure 7A). Transfection of mouse embryos with poFUT1 siRNA or poFUT1 cDNA inhibited and promoted the expression of poFUT1 and the capacity for invasion, respectively (Figure 7B). Most importantly, that epiregulin reduced E‐cadherin expression, whereas it increased N‐cadherin expression in mouse embryos. The invasion potential cells migrated far from the embryo centre (Figure 7C). Then, we observed that epiregulin increased the expression of HLA‐G, which is an EVT trophoblast cell marker (Figure 7D). Then, we further analysis the role of epiregulin and poFUT1 on embryo implantation rate. Mouse morulae or blastocysts were incubated with epiregulin antibody or poFUT1 siRNA for 48 hours; then, the embryos were transferred into pseudocyesis mice uterus, and the mice were sacrificed at PD8 to analyse the embryo implantation rate. The statistical results showed that poFUT1 siRNA suppressed the embryo implantation rate compared with the control group, and epiregulin antibody blockade group decreased the embryo implantation rate compared with control group (Figure 7E,F). These findings suggest that epiregulin and poFUT1 are essential for promoting embryo invasion and successful embryo implantation in mice.

## 4 DISCUSSION

During embryo implantation, the transformation from the trophoblastic epithelial‐like cytotrophoblast (CTB) to the mesenchymal‐like EVT is an essential process that facilitates embryo invasion of the uterine epithelium. The CTB‐EVT conversion process has been described as “pseudo‐EMT.”^29^, ^30^, ^31^ Many regulators affect EVT migration and invasion, such as hormones, cytokines and chemokines. For example, gonadotropin induces trophoblast cell migration and invasion by EMT upregulation.^32^ MMP14 activation is critical for EMT and migration by regulating the levels of cadherins in the cells.^33^ Autocrine TGFβ/TGFβR signalling can stimulate the EMT in the cancer cells.^34^ Our results also provided the evidence that poFUT1, epiregulin and uPA were expressed at lower levels in trophoblast from abortion patients (Figure 1A) than normal pregnant women, and decreased level of these molecules could hamper embryo implantation by interfering the trophoblast EMT process.

Epiregulin is secreted by early human placenta and uterine glands.^9^, ^10^, ^11^ Epiregulin is a member of the EGF family, which are involved in many physiological and pathological processes, such as oocyte maturation, cutaneous wound healing and vascular remodelling.^7^ Epiregulin usually plays roles by binding its ligands. EGF ligands include EGF, TGFα, HB‐EGF, betacellulin (BTC), amphiregulin (AREG), epiregulin (EREG) and epigen (EPGN).^35^ The EGF family regulates the processes of trophoblast differentiation, trophoblast‐uterine communication and trophoblast adhesion/invasion by autocrine, paracrine and juxtacrine mechanisms.^36^, ^37^ EGF and HB‐EGF are expressed in the decidua, while facilitating trophoblast invasion and villous explants outgrowth.^38^, ^39^ EGF has been shown to increase MMP‐2 and MMP‐9 activity in trophoblastic cells, resulting in cell invasion.^40^ Betacellulin, amphiregulin and epiregulin are highly expressed in the chorionic villi at term compared with preeclampsia and preterm labour.^41^ EGF‐like peptides contribute to oocyte developmental competence. In the current study, we further found that a lower level of epiregulin was associated with miscarriage, and epiregulin facilitated the invasion and EMT in trophoblastic cells (Figure 2).

The glycan structures, including N‐glycans and O‐glycans, have been described to affect cell migration, invasion and EMT process. B4GALT3 has been found to suppress EVT invasion in the late stages of pregnancy.^42^ Overexpression of GALNT2 (O‐glycosyltransferase) enhances the O‐glycosylation of β1 integrin and suppresses EVT cell invasion.^43^ GALNT3 maintains the epithelial state in trophoblast stem cells.^44^ FUT8 increases the migration and invasion of trophoblast cells.^45^ O‐fucose glycans are essential for Notch signalling and embryonic development in mice.^46^ We previously found that progesterone increased the expression of poFUT1 and promoted the proliferation and adhesion of JAR cells. In the current study, the results showed that epiregulin also increased the expression of poFUT1 through the c‐Fos/c‐Jun transcription factor in trophoblast cells (Figure 3). Epiregulin increased the biosynthesis of O‐fucosylation in trophoblastic cells (Figure 3I,J), indicating that it participates in the regulation of trophoblast cell invasion and EMT process.

Glycosylation of the proteins usually regulates the biological functions and controls cellular phenotypes. Many EGF repeats are modified at evolutionarily conserved consensus sites by an unusual form of O‐fucose. Mammalian proteins are modified with O‐fucose, including Notch1, uPA, nidogen‐2 and neurocan core protein. uPA contains an EGF repeat domain and is fucosylated on Thr^18^.^47^, ^48^ Activated uPA converts plasminogen into plasmin, which in turn activates MMPs, and then degrades ECM. EMT and cell motile behaviour, which are induced by the increased expression of ECM‐degrading enzymes, such as uPA and MMPs, enhance tumour cell invasion. Elevated uPAR expression in tumour cells correlates with poor prognosis, invasion and lymph node metastasis in squamous cell carcinoma.^49^ TGFβ enhances uPA expression, leading to a greater invasiveness of MDA‐MB‐231 cells.^50^ Conversely, downregulation of uPA by miR‐23b decreased migration ability of human hepatocellular carcinoma cells.^51^ Regarding cellular surface glycoproteins, we decided to determine whether the poFUT1 or O‐fucose‐modified proteins were necessary for invasion of trophoblastic cells. uPA is present in the EVT of the human placenta, and the binding of uPA and uPAR at the migration frontier of EVT cells suggests that uPA/uPAR plays a role during EVT cells' invasion into the uterine decidua. We found that increased poFUT1 promoted the biosynthesis of uPA (Figure 4E). Significantly, when transfected with poFUT1 cDNA, trophoblast cells activated the PI3K/Akt signalling pathway and increased the secretion of MMP9, which degraded the ECM (Figure 5) and promoted EMT processing. These results suggest that poFUT1 upregulates biosynthesis of O‐fucosylation and the binding of uPA and uPAR, further facilitating EMT.

**Figure 8:**
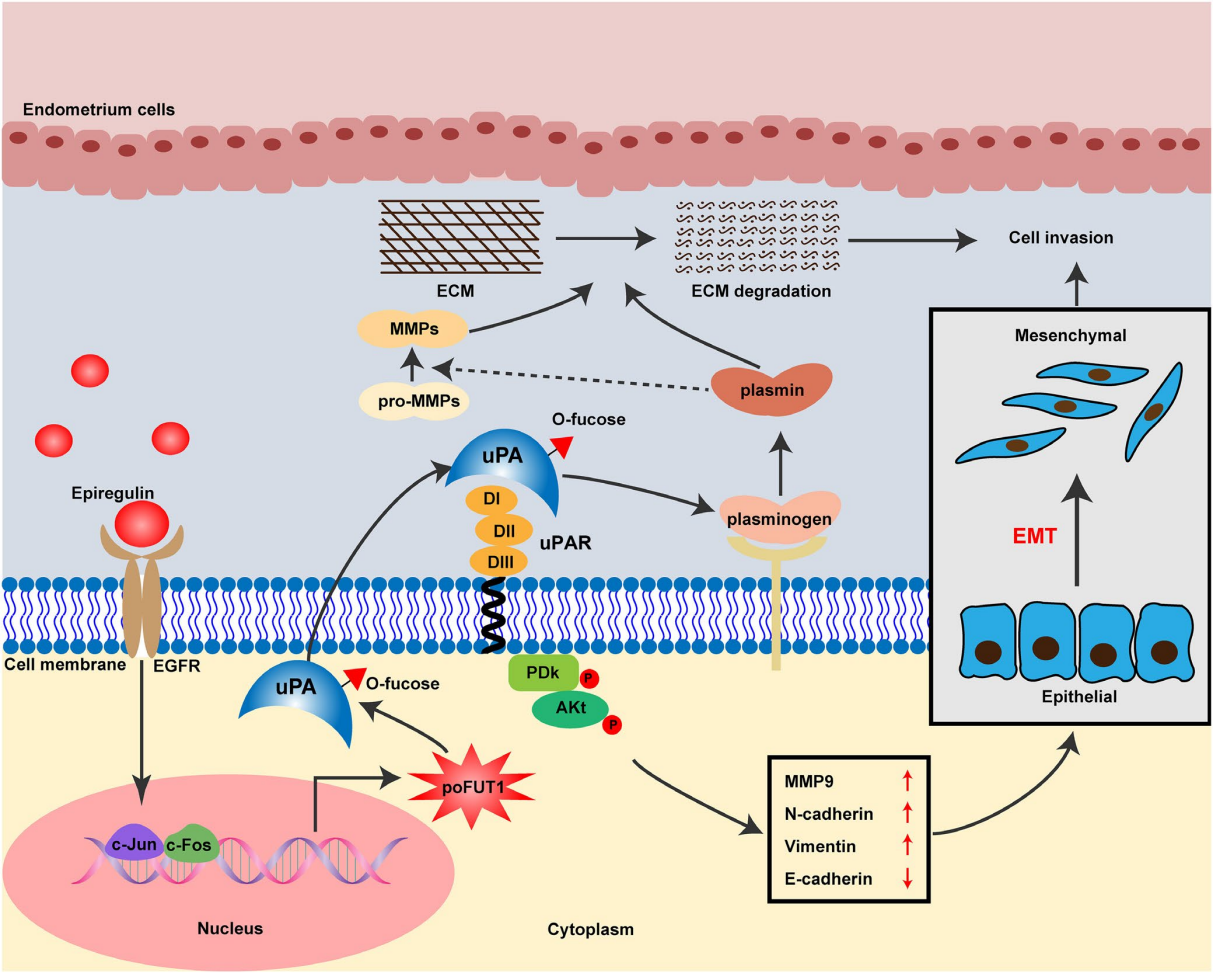
The schematic illustration summarizes the epiregulin promoted trophoblast cells invasion by targeting poFUT1 and O‐fucosylation on uPA, which stimulated PI3K/Akt signalling pathway and MMP 9 activity. poFUT1, protein O‐fucosyltransferase 1; uPA, urokinase‐type plasminogen activator

A good deal of knowledge is currently available on the molecular mechanisms of miscarriage. Disordered mechanisms may produce failed embryo implantation. Our study revealed that epiregulin increased poFUT1 and O‐fucosylation on uPA, which further activated the PI3K/Akt signalling pathway and caused trophoblast invasion and EMT (Figure 8). Our findings are essential in that they identify early markers (poFUT1 and epiregulin) of miscarriage in serum and trophoblastic cells, which may serve as new biomarkers for clinical diagnosis and provide the foundation for strategies of infertility treatment.

## CONFLICT OF INTEREST

The authors declare that they have no competing interests.

## AUTHOR CONTRIBUTIONS

Shuai Liu and Qiu Yan designed the experiments. Xinyuan Cui wrote the manuscript. Xinyuan Cui, Hao Wang, Yaqiu Li and Tianhong Chen performed the experiments. Xinyuan Cui performed statistical analysis. All authors reviewed the manuscript.

## Data Availability

The data that support the findings of this study are available from the corresponding author upon reasonable request.

## References

[1] Paul ABM , Sadek ST , Mahesan AM . The role of microRNAs in human embryo implantation: a review. J Assist Reprod Genet. 2019;36:179‐187. 30315515 10.1007/s10815-018-1326-y PMC6420523

[2] Salamonsen LA , Evans J , Nguyen HP , Edgell TA . The microenvironment of human implantation: determinant of reproductive success. Am J Reprod Immunol. 2016;75:218‐225. 26661899 10.1111/aji.12450

[3] Chaudhary P , Malhotra SS , Babu GS , Sobti RC , Gupta SK . HGF promotes HTR‐8/SVneo cell migration through activation of MAPK/PKA signaling leading to up‐regulation of WNT ligands and integrins that target beta‐catenin. Mol Cell Biochem. 2019;453:11‐32. 30136190 10.1007/s11010-018-3428-3

[4] Huppertz B , Weiss G , Moser G . Trophoblast invasion and oxygenation of the placenta: measurements versus presumptions. J Reprod Immunol. 2014;101‐102:74‐79. 10.1016/j.jri.2013.04.003 23747129

[5] Lunghi L , Ferretti ME , Medici S , Biondi C , Vesce F . Control of human trophoblast function. Reprod Biol Endocrinol. 2007;5:6. 17288592 10.1186/1477-7827-5-6 PMC1800852

[6] Cartwright JE , Fraser R , Leslie K , Wallace AE , James JL . Remodelling at the maternal‐fetal interface: relevance to human pregnancy disorders. Reproduction. 2010;140:803‐813. 20837731 10.1530/REP-10-0294

[7] Riese DJ 2nd , Cullum RL . Epiregulin: roles in normal physiology and cancer. Semin Cell Dev Biol. 2014;28:49‐56. 24631357 10.1016/j.semcdb.2014.03.005 PMC4037385

[8] Harris RC , Chung E , Coffey RJ . EGF receptor ligands. Exp Cell Res. 2003;284:2‐13. 12648462 10.1016/s0014-4827(02)00105-2

[9] Das SK , Das N , Wang J , et al. Expression of betacellulin and epiregulin genes in the mouse uterus temporally by the blastocyst solely at the site of its apposition is coincident with the "window" of implantation. Dev Biol. 1997;190:178‐190. 9344537 10.1006/dbio.1997.8694

[10] Song H , Lim H , Das SK , Paria BC , Dey SK . Dysregulation of EGF family of growth factors and COX‐2 in the uterus during the preattachment and attachment reactions of the blastocyst with the luminal epithelium correlates with implantation failure in LIF‐deficient mice. Mol Endocrinol. 2000;14:1147‐1161. 10935540 10.1210/mend.14.8.0498

[11] Toyoda H , Komurasaki T , Uchida D , Morimoto S . Distribution of mRNA for human epiregulin, a differentially expressed member of the epidermal growth factor family. Biochem J. 1997;326(Pt 1):69‐75. 9337852 10.1042/bj3260069 PMC1218638

[12] Costache M , Cailleau A , Fernandez‐Mateos P , Oriol R , Mollicone R . Advances in molecular genetics of alpha‐2‐ and alpha‐3/4‐fucosyltransferases. Transfus Clin Biol. 1997;4:367‐382. 9269717 10.1016/s1246-7820(97)80042-0

[13] Shi S , Stanley P . Protein O‐fucosyltransferase 1 is an essential component of Notch signaling pathways. Proc Natl Acad Sci USA. 2003;100:5234‐5239. 12697902 10.1073/pnas.0831126100 PMC154328

[14] Lira‐Navarrete E , Valero‐Gonzalez J , Villanueva R , et al. Structural insights into the mechanism of protein O‐fucosylation. PLoS ONE. 2011;6:e25365. 21966509 10.1371/journal.pone.0025365 PMC3180450

[15] Takeuchi H , Wong D , Schneider M , et al. Variant in human POFUT1 reduces enzymatic activity and likely causes a recessive microcephaly, global developmental delay with cardiac and vascular features. Glycobiology. 2018;28:276‐283. 29452367 10.1093/glycob/cwy014 PMC6057529

[16] Carson DD . The glycobiology of implantation. Front Biosci. 2002;7:d1535‐d1544. 12045005 10.2741/A858

[17] Jones CJ , Aplin JD . Glycosylation at the fetomaternal interface: does the glycocode play a critical role in implantation? Glycoconj J. 2009;26:359‐366. 18677581 10.1007/s10719-008-9152-6

[18] Zhang Y , Liu S , Liu Y , Wang Z , Wang X , Yan Q . Overexpression of fucosyltransferase VII (FUT7) promotes embryo adhesion and implantation. Fertil Steril. 2009;91:908‐914. 18402946 10.1016/j.fertnstert.2007.12.012

[19] Gu J , Sui LL , Cui D , Ma YN , Zhu CY , Kong Y . Effects of LeY glycan expression on embryo implantation. Eur Rev Med Pharmacol Sci. 2016;20:3327‐3335. 27608889

[20] Mahmood N , Mihalcioiu C , Rabbani SA . Multifaceted role of the urokinase‐type plasminogen activator (uPA) and its receptor (uPAR): diagnostic, prognostic, and therapeutic applications. Front Oncol. 2018;8:24. 29484286 10.3389/fonc.2018.00024 PMC5816037

[21] Rabbani SA , Mazar AP , Bernier SM , et al. Structural requirements for the growth factor activity of the amino‐terminal domain of urokinase. J Biol Chem. 1992;267:14151‐14156. 1321137

[22] McMahon BJ , Kwaan HC . Components of the plasminogen‐plasmin system as biologic markers for cancer. Adv Exp Med Biol. 2015;867:145‐156. 26530365 10.1007/978-94-017-7215-0_10

[23] Hosseini N , Khoshnazar A , Saidijam M , et al. Zerumbone suppresses human colorectal cancer invasion and metastasis via modulation of FAk/PI3k/NFkappaB‐uPA pathway. Nutr Cancer. 2019;71:159‐171. 30650987 10.1080/01635581.2018.1540719

[24] Chen JK , Peng SF , Lai KC , et al. Fistein suppresses human osteosarcoma U‐2 OS cell migration and invasion via affecting FAK, uPA and NF‐kB signaling pathway in vitro. Vivo. 2019;33:801‐810. 10.21873/invivo.11542 PMC6559886 31028200

[25] Martinez‐Soto JC , Landeras J , Mollá M , et al. Total urokinase‐type plasminogen activator (uPA) levels in seminal plasma are associated with positive assisted reproductive technology outcomes. J Assist Reprod Genet. 2018;35:1091‐1101. 29572694 10.1007/s10815-018-1164-y PMC6030003

[26] Uszynski M , Perlik M , Uszynski W , Zekanowska E . Urokinase plasminogen activator (uPA) and its receptor (uPAR) in gestational tissues; measurements and clinical implications. Eur J Obstet Gynecol Reprod Biol. 2004;114:54‐58. 15099871 10.1016/j.ejogrb.2003.12.006

[27] Wang XJ , Zhou ZY , Xu YJ . [Changes of plasma uPA and TGF‐beta1 in patients with preeclampsia]. Sichuan Da Xue Xue Bao Yi Xue Ban. 2010;41:118‐120. 20369484

[28] Guibourdenche J , Leguy MC , Tsatsaris V . [Biology and markers of preeclampsia]. Ann Biol Clin (Paris). 2013;71:79‐87. 10.1684/abc.2013.0903 24235331

[29] Kalluri R , Weinberg RA . The basics of epithelial‐mesenchymal transition. J Clin Invest. 2009;119:1420‐1428. 19487818 10.1172/JCI39104 PMC2689101

[30] Duzyj CM , Buhimschi IA , Motawea H , et al. The invasive phenotype of placenta accreta extravillous trophoblasts associates with loss of E‐cadherin. Placenta. 2015;36:645‐651. 25904157 10.1016/j.placenta.2015.04.001

[31] E Davies J , Pollheimer J , Yong HE , et al. Epithelial‐mesenchymal transition during extravillous trophoblast differentiation. Cell Adh Migr. 2016;10:310‐321. 27070187 10.1080/19336918.2016.1170258 PMC4951171

[32] Feng D , Zhao T , Yan K , et al. Gonadotropins promote human ovarian cancer cell migration and invasion via a cyclooxygenase 2‐dependent pathway. Oncol Rep. 2017;38:1091‐1098. 28677781 10.3892/or.2017.5784

[33] Garmon T , Wittling M , Nie S . MMP14 regulates cranial neural crest epithelial‐to‐mesenchymal transition and migration. Dev Dyn. 2018;247:1083‐1092. 30079980 10.1002/dvdy.24661 PMC6150824

[34] Hao Y , Baker D , Ten Dijke P . TGF‐beta‐mediated epithelial‐mesenchymal transition and cancer metastasis. Int J Mol Sci. 2019;20:2767. 10.3390/ijms20112767 PMC6600375 31195692

[35] Freed DM , Bessman NJ , Kiyatkin A , et al. EGFR ligands differentially stabilize receptor dimers to specify signaling kinetics. Cell. 2017;171:683‐695.e618. 28988771 10.1016/j.cell.2017.09.017 PMC5650921

[36] Guzeloglu‐Kayisli O , Kayisli UA , Taylor HS . The role of growth factors and cytokines during implantation: endocrine and paracrine interactions. Semin Reprod Med. 2009;27:62‐79. 19197806 10.1055/s-0028-1108011 PMC3107839

[37] Fritz R , Jain C , Armant DR . Cell signaling in trophoblast‐uterine communication. Int J Dev Biol. 2014;58:261‐271. 25023692 10.1387/ijdb.140011da PMC10411524

[38] Hofmann GE , Scott RT Jr , Bergh PA , Deligdisch L . Immunohistochemical localization of epidermal growth factor in human endometrium, decidua, and placenta. J Clin Endocrinol Metab. 1991;73:882‐887. 1890159 10.1210/jcem-73-4-882

[39] Bass KE , Morrish D , Roth I , et al. Human cytotrophoblast invasion is up‐regulated by epidermal growth factor: evidence that paracrine factors modify this process. Dev Biol. 1994;164:550‐561. 8045351 10.1006/dbio.1994.1223

[40] Staun‐Ram E , Goldman S , Gabarin D , Shalev E . Expression and importance of matrix metalloproteinase 2 and 9 (MMP‐2 and ‐9) in human trophoblast invasion. Reprod Biol Endocrinol. 2004;2:59. 15294019 10.1186/1477-7827-2-59 PMC516041

[41] Armant DR , Fritz R , Kilburn BA , et al. Reduced expression of the epidermal growth factor signaling system in preeclampsia. Placenta. 2015;36:270‐278. 25589361 10.1016/j.placenta.2014.12.006 PMC4331205

[42] Liao WC , Liu CH , Chen CH , et al. beta‐1,4‐Galactosyltransferase III suppresses extravillous trophoblast invasion through modifying beta1‐integrin glycosylation. Placenta. 2015;36:357‐364. 25659296 10.1016/j.placenta.2015.01.008

[43] Liao W‐C , Chen C‐H , Liu C‐H , et al. Expression of GALNT2 in human extravillous trophoblasts and its suppressive role in trophoblast invasion. Placenta. 2012;33:1005‐1011. 23117232 10.1016/j.placenta.2012.08.007

[44] Raghu D , Mobley RJ , Shendy NAM , Perry CH , Abell AN . GALNT3 maintains the epithelial state in trophoblast stem cells. Cell Rep. 2019;26:3684‐3697. 30917321 10.1016/j.celrep.2019.02.093 PMC6501849

[45] Yu M , Cui X , Wang H , et al. FUT8 drives the proliferation and invasion of trophoblastic cells via IGF‐1/IGF‐1R signaling pathway. Placenta. 2019;75:45‐53. 30712666 10.1016/j.placenta.2018.11.005

[46] Batista F , Lu L , Williams SA , Stanley P . Complex N‐glycans are essential, but core 1 and 2 mucin O‐glycans, O‐fucose glycans, and NOTCH1 are dispensable, for mammalian spermatogenesis. Biol Reprod. 2012;86:179. 22492969 10.1095/biolreprod.111.098103 PMC3386147

[47] Rampal R , Luther KB , Haltiwanger RS . Notch signaling in normal and disease States: possible therapies related to glycosylation. Curr Mol Med. 2007;7:427‐445. 17584081 10.2174/156652407780831593

[48] Harris RJ , Spellman MW . O‐linked fucose and other post‐translational modifications unique to EGF modules. Glycobiology. 1993;3:219‐224. 8358148 10.1093/glycob/3.3.219

[49] Bacchiocchi R , Rubini C , Pierpaoli E , et al. Prognostic value analysis of urokinase‐type plasminogen activator receptor in oral squamous cell carcinoma: an immunohistochemical study. BMC Cancer. 2008;8:220. 18673553 10.1186/1471-2407-8-220 PMC2527016

[50] Shiou SR , Datta PK , Dhawan P , et al. Smad4‐dependent regulation of urokinase plasminogen activator secretion and RNA stability associated with invasiveness by autocrine and paracrine transforming growth factor‐beta. J Biol Chem. 2006;281:33971‐33981. 16959768 10.1074/jbc.M607010200

[51] Salvi A , Sabelli C , Moncini S , et al. MicroRNA‐23b mediates urokinase and c‐met downmodulation and a decreased migration of human hepatocellular carcinoma cells. FEBS J. 2009;276:2966‐2982. 19490101 10.1111/j.1742-4658.2009.07014.x

